# The Prevalence of Hypomagnesaemia in Pediatric Patients with Mitral Valve Prolapse Syndrome and the Effect of Mg Therapy

**Published:** 2012-09-15

**Authors:** Hamid Amoozgar, Hashem Rafizadeh, Gholamhossein Ajami, Mohammad Borzoee

**Affiliations:** 1 Division of Pediatric Cardiology, Shiraz University of Medical Sciences, Shiraz, IR Iran

**Keywords:** Dysautonomia, Echocardiography, Hypomagnesaemia, Mitral Valve Prolapse, Mitral Valve Prolapse Syndrome

## Abstract

**Background:**

A paucity of data exists regarding the prevalence and relationship of hypomagnesaemia with clinical symptoms of mitral valve prolapse (MVP) in pediatric patients.

**Objective:**

In this study we evaluated the prevalence of magnesium (Mg) deficiency in pediatric patients with MVP syndrome and attempted to clarify the effect of Mg therapy on alleviating their symptoms.

**Methods:**

The present study was conducted from April 2010 to January 2012, and included 230 patients (90 males and 140 females) with symptoms of mitral valve prolapse and mean age of 11.6±3.66. Serum magnesium (Mg) level less than 1.5 mg/dl was defined as hypomagnesaemia. Patients with 2 mm leaflet displacement and maximum leaflet thickness of 5 mm in echocardiography were considered to have classic MVP, while those with leaflet thickness less than 5 mm were considered as non-classic MVP. Patients with hypomagnesaemia were orally treated with 4.5 mg/kg/day Mg chloride for 5 weeks followed by re-evaluation of symptoms of chest pain, palpitation, fatigue and dyspnea.

**Results:**

Hypomagnesaemia was found in 19 (8.2 %) of 230 patients with mitral valve prolapse. The re-evaluation of patients with Hypomagnesaemia after 5 weeks of Mg therapy, showed statistically significant relief of chest pain (P=0.01). However, no significant changes was detected in regard to palpitation (P=0.06), fatigue (P= 0.5) and dyspnea (P=0.99).

**Conclusion:**

This study revealed that the prevalence of hypomagnesaemia in pediatric patients with mitral valve prolapse is relatively low compared to adults, but treatment with oral Mg in patient with hypomagnesaemia decreases chest pain.

## Introduction

Mitral valve prolapse (MVP) is a relatively common medical problem with many controversies and confusions among physician concerning its diagnosis and management. MVP represents a heart valve with an abnormal displacement of one or both mitral valve leaflets during systole, and is routinely diagnosed as a faint heart “click” or murmur. It is usually a benign condition, though it is related to a confusing array of seemingly unrelated symptoms, from chest pain, shortness of breath to panic attacks. Since reduction in risk of rheumatic valvular disease, MVP has become the most common valvular disorder in developed countries. The prevalence of MVP was reported between 2 to 5 percent among different populations (1- 3).

Previously, a set of symptoms known as dysautonomia was thought to occur in association with MVP which was collectively called Mitral Valve Prolapse Syndrome (4,5).

Among several nutrients involved in mitral valve prolapse symptoms, magnesium (Mg) is probably the most significant element. Mg deficiencies are associated with migraine headaches, common in people with epilepsy and also reported in relation to MVP. There are few data about Mg deficiency in pediatric patients with MVP (6). This prospective study was carried out to evaluate the prevalence of Mg deficiency in pediatric patients with MVP and attempted to clarify the impact of Mg therapy on reduction of symptoms in pediatric patients with hypomagnesaemia.

## Patients and Methods

This study was conducted from April 2010 to January 2012, and included 230 consecutive patients with symptoms of mitral valve prolapse syndrome referred to Emam Reza Clinic ,the main pediatric cardiology center affiliated to Shiraz University of Medical Sciences. The patients were examined in supine, sitting, and standing positions. A midsystolic or multiple clicks followed by a midsystolic to late systolic murmur at the apex of the left ventricle over the mitral area was considered as sign of MVP. The MVP in patients was characterized by any combination of palpitations, dyspnea, exercise intolerance, dizziness, syncope, panic, anxiety disorders, numbness or tingling, and skeletal abnormalities in addition to prolapsing mitral valve leaflets.

Two-dimensional, M-mode and pulse Doppler echocardiography were done with a General Electric Vivid 3 echocardiographic machine (General Electric, Vingmed, Horten, Norway) and a 3 and 5-MHz probe. M-mode criteria for the diagnosis of MVP were at least 3 mm holosystolic displacement or a minimum of 2 mm late systolic displacement of the mitral valve leaflet behind the C-D line. Two-dimensional echocardiographic criteria of 2 mm leaflet displacement and maximum 5 mm leaflet thickness were considered as classic MVP, while those with 2 mm leaflet displacement and leaflet thickness less than 5 mm were classified as non-classic MVP (7-11).

Mg was measured by endpoint colorimetric method (direct and chromogene xylidyl blue). Serum Mg level less than 1.5 mg/dl was defined as hypomagnesaemia (12).

Patient with hypomagnesaemia, were treated with oral Mg chloride 4.5 mg/kg/day for 5 weeks and re-evaluated by a questioner for symptoms of chest pain, palpitation, lethargy and dyspnea (12).

Statistical analysis included Student’s t-test to compare continuous variables, Pearson correlation for evaluation of correlation between hypomagnesaemia with various clinical parameters, and Mann–Whitney test to compare non parametric variables. The data were expressed as the mean ± standard deviation. SPSS version 15 statistical software was used for all the statistical tests and a p value of < 0.05 was considered significant.

## Results

Of 230 patients, 90 were males and 140 were females (male to female ratio 0.64). The patients aged from 2 to18 years with an average 11.6±3.66 years and average weight of 35.2±11.41 kg.

Mid systolic click was heard on physical examination of 186 (80%) patients. Positive tomb sign was seen in 194 (84%) patients.

The mean serum Mg was 2.21±0.47mg/dL. The serum Mg levels in patients were shown in Figure. 1. Of 230 patients 19 (8.2 %) cases had serum Mg lower than 1.5mg/dL. There were no statistically significant differences between males and females in regard to age (P=0.17), weight (P=0.51) and serum Mg level (2.26±0.53 vs 2.18±0.43, p=0.36). Also no statistically significant correlation was found between serum Mg (P=0.32) and age and weight (P= 0.21)of patients.

Echocardiography revealed mitral regurgitation in 119 (51.7 %) out of 230 patients. In this context, no statistically significant difference was found between them and other patients in relation to age (P=0.93), weight (P=0.46) and value of serum Mg (2.26±0.44 vs 2.15±049, P=0.20)

Also of 230 patients 38 (16.5 %) subjects had prolapsing tricuspid, which was not significantly differenet from other patients in terms of age (P=0.17), weight (P=0.29) and serum level of Mg (2.21±048 vs 2.16±0.43, P=0.68).

Of 230 patients, classic MVP was present in 34 (14.7 %) cases, which was statistically significant in lower age (P=0.01) and weight (P=0.03) compared with no-nclassic MVP. However, there was no statistically significant difference in serum level of Mg with respect to the patients without classic sign of MVP (P=0.79). Three patients with classic MVP had hypomagnesaemia (8.8%) (Table 1). There was no statistically significant difference between Males and females, regarding mitral regurgitation, tricuspid prolapse and classic MVP with respective values of 45.7% vs 56.2% P= 0.26, 10.9% vs 16.4% (P=0.39) and 10% vs 13% (P =0.65).

**Table 1:** Comparison between Patients with and Without Classic Mitral Valve Prolapse

Variable	Classic	Non-classic	P value
**Age**	9.2±4.21	12.5±3.53	0.01
**Weight( Kg)**	27.11±11.37	37.23±11.99	0.03
**Magnesium(mg/dL)**	2.23±0.27	2.23±0.53	0.79

In 19 patients with hypomagnesaemia re-evaluated after 5 weeks of Mg therapy, chest pain decreased significantly per week per patient (P=0.015) ,but palpitation (P=0.06), fatigue (P=0.5) and dyspnea (P=0.99) were unaffected.

## Discussion

Low levels of magnesium can cause some symptoms similar to dysautonomia and people receiving magnesium experienced a significant reduction in dysautonomic symptoms, such as chest pain, palpitations, anxiety, and shortness of breath (6). Considering the data available in adults (6), the present investigation attempted to study the prevalence of hypomagnesaemia and the effect of magnesium supplement therapy on children with MVP syndrome. This study showed hypomagnesaemia in 8.2 % of pediatric patients with MVP, and significant reduction in chest pain by Mg therapy. The prevalence of hypomagnesaemia in our study was lower than that of adults (6). In a report by Lichodziejewska et.al, on 141 adult patients with MVP, a significant number of patients exhibited low serum Mg as compared to 40 healthy controls group (60% versus 5 %), and concluded that many patients with severe MVP symptoms had low serum Mg, and supplementation of this ion led to improvement in most symptoms along with a decrease in catecholamine excretion (6).

In idiopathic MVP most patients describe a great variety of symptoms. Idiopathic MVP may be a form of latent tetany due to Mg deficiency. The prevalence, latent nature, and symptomatology of these two conditions appear to be very similar. The clinical pictures of idiopathic MVP and latent tetany are superimposed and are related to Mg deficiency. The Mg deficit in these patients has various causes like insufficient Mg intake, depletion of Mg provoked by stress, excess coffee consumption, corticosteroids or catecholamine excess. The high prevalence of this defect in women is mostly related to the ovarian hormones. Constitutional factors such as the HLA-Bw35 antigen and the type A of personality may also be the cause of Mg depletion (13,14).

Latent tetany due to chronic Mg deficiency occurs in over 85% of MVP cases in adults, and MVP complicates 26% of latent tetany. Mg deficiency can explain many clinical features of the MVP syndrome which are not easily explained in terms of genetics. Mg deficiency may underlie the mechanism by which fibroblasts degrade defective collagen, increase in circulating catecholamines, predisposition to cardiac arrhythmias, thromboembolic phenomena and dysregulation of the immune and autonomic nervous systems (15). The relief of MVP symptoms by MG therapy observed in the present investigation was consistent with that of another study (15).

As there are a few studies carried out on children, Bobkowski W, et.al, reported a marked decrease in Mg concentration in symptomatic children with MVP, compared to asymptomatic patients (P<0.001). In this context, a significantly positive correlation was found between Mg concentration and high frequency power components and significant negative correlation between Mg concentration and low incidence of parameters as well as low to high frequency ratio of frequency domain analysis of heart rate in daytime and nighttime). They concluded that in children with MVP the autonomic changes including reduced parasympathetic tone and sympathetic predominance were correlated with decreased serum Mg concentration and clinical symptoms. Also Mg supplementation seemed to be beneficial in symptomatic children with MVP (16).

In a study by Syedmoradi L et.al, in Iranian urban population, the prevalence of hypomagnesaemia was 4.6% which was more prevalent in females (6.0%) than in males (3.2%, P<0.05) (17).

The mean age of the patients in our study was 11.6±3.66 years. Using two-dimensional echocardiography in 4,328 children aged from 1 day to 15 years, the incidence of MVP was reported to rise with increasing age, although MVP was not observed in children aged from 1 to 28 days. However, the incidence of MVP was 0.25%, 2.1% and 5.1% in children aged from 6 to 18 months, 6 to 7 years, and 12 to 15 years respectively. (18).

The ratio of females to males was 1.58/1 in this study. Framingham used two-dimensional echocardiography to evaluate MVP in patients, and overall found a prevalence of 2.4 percent which was similar in both men and women. (1). In another study using clinical auscultation, MVP was found in 331 (5.37%) children including 175 boys and 156 girls and aged from 2 months to 21 years. (15). Echocardiographic study of 4136 young adults, aged from 23 to 35 years, showed a prevalence of 0.6 percent MVP with similar frequency in both men and women and in black and white subjects. The reported incidence and age and gender distribution differ significantly from study to study, which was due to a lack of definite criteria for diagnosis of MVP, differences in study design, and some selection bias. Of 230 patients, classic MVP was found in 34(14.7%) cases, that differed significantly from patients without classic sign of MVP regarding lower age (P=0.01) and lower weight (P=0.03). Freed, LA, et al. detected classic MVP in 1.3% of their studied population (1). Our findings indicated that the serum level of Mg and frequency of hypomagnesaemia were not significantly different in patients without classic sign of MVP (P>0.05).

According to our study, males and females did not exhibit any statistically significant difference in mitral regurgitation (45.7% vs 56.2%, P= 0.26), tricuspid prolapse (10.9% vs 16.4%, P=0.39), and classic MVP. In this context, Freed, LA et al. reported severe mitral regurgitation in 7% of patients with classic MVP (1).

A double-blind, placebo-controlled-crossover trial is needed to explore the effect of hypomagnesaemia in management of chest pain in children with MVP syndrome. Also the use of a standard pain scale might be useful for assessment of response to treatment.

The prevalence of hypomagnesaemia in pediatric patients with MVP is not as high as in adults. Age, gender, mitral regurgitation and classic sign were not correlated with hypomagnesaemia in pediatric patients with MVP. However as in adults, oral Mg therapy decreased chest pain in pediatric patients with hypomagnesaemia.
